# How personality became treatable: The mutual constitution of clinical knowledge and mental health law

**DOI:** 10.1177/0306312712457722

**Published:** 2013-02

**Authors:** Martyn Pickersgill

**Affiliations:** Centre for Population Health Sciences, University of Edinburgh, UK

**Keywords:** law, mental health, personality disorder, psychiatry, psychology, psychopathy

## Abstract

In recent years, personality disorders – psychiatric constructs understood as enduring dysfunctions of personality – have come into ever-greater focus for British policymakers, mental health professionals and service-users. Disputes have focussed largely on highly controversial attempts by the UK Department of Health to introduce mental health law and policy (now enshrined within the 2007 Mental Health Act of England and Wales). At the same time, clinical framings of personality disorder have dramatically shifted: once regarded as untreatable conditions, severe personality disorders are today thought of by many clinicians to be responsive to psychiatric and psychological intervention. In this article, I chart this transformation by means of a diachronic analysis of debates and institutional shifts pertaining to both attempts to change the law, and understandings of personality disorder. In so doing, I show how mental health policy and practice have mutually constituted one another, such that the aims of clinicians and policymakers have come to be closely aligned. I argue that it is precisely through these reciprocally constitutive processes that the profound reconfiguration of personality disorder from being an obdurate to a plastic condition has occurred; this demonstrates the significance of interactions between law and the health professions in shaping not only the State’s management of pathology, but also perceptions of its very nature.

In the UK, as well as many other nations, psychiatric disorders relating to antisocial and criminal behaviour are objects of public, political and medical concern. Particular interest has been focused upon the constructs of antisocial personality disorder (ASPD) and psychopathic personality disorder (psychopathy).^1^ Meditations on disordered personalities date back to at least the 19th century (Berrios, 1993), and the ethical and clinical basis of maintaining personality disorders as the concern of the mental health professions has long been debated (Eastman and Starling, 2006). In part, this is a consequence of the fact that conditions such as ASPD and psychopathy are framed as global personality dysfunctions, rather than as discrete disorders (such as schizophrenia or depression). Individuals regarded as personality disordered – especially those who have committed criminal offences – transgress a range of boundaries: between normality and pathology, victim and perpetrator, and mental patient and criminal. These transgressions engender important questions about how the State can and should manage persons living under the label of personality disorder, questions that continue to resist easy answers.

In the UK, the increasing prominence of personality disorders in general, and ASPD and psychopathy in particular, is both a consequence of the perceived costs to society resulting from the actions of personality disordered individuals, and a result of the drafting of controversial mental health legislation (debates about which often focused on personality disorders (Pilgrim, 2007)). In 1999, the Government began to formulate plans for rewriting the 1983 Mental Health Act (MHA) of England and Wales.^2^ These plans captured the attention of health professionals, who were keenly aware of the potential for new policy to reshape practice. However, the possible effects of new governance (and the clinical discourses that revolved around it) on framings of pathology were less widely appreciated. As debates over the MHA unfolded, personality disorder came to be regarded in new ways. Traditionally, ASPD and psychopathy have been thought to be resistant to clinical intervention; this was the dominant framing of these conditions even into the late 1990s. Yet, as debates on the MHA played out, framings of personality disorders came gradually, but profoundly, to position these disorders as plastic rather than obdurate constructs: in effect, they became treatable.

In this article, I historicize these discourses; in particular, I seek to demonstrate the reciprocal shaping of clinical and legal debates that reconfigured understandings of personality disorders such that many clinicians came to regard them as being amenable to treatment interventions. Methodologically, my research examines personality disorder (particularly, ASPD) through an analysis of its framing within mental health policy debates and clinical discourse. I analysed all issues of the *British Journal of Psychiatry* (*BJP*) from 1950 to 2012 for articles, editorials and correspondence on personality disorder and mental health law, as well as other key papers, such as those that were frequently referenced in the *BJP* articles I analysed. I paid special attention to writings from the late 1990s onwards, which were often part of a wider conversation around proposals to introduce a new MHA. I also inspected policy documents from the Department of Health (DH), which pertained to the re-writing of the 1983 MHA and the instatement of new services for personality disorder, and I read a wide range of commentaries by lawyers on the legal changes taking place. I consulted these materials in order to gain access to the views of the range of experts who had a stake in the legal and clinical innovations taking place in forensic mental health. I also took the opportunity to attend conferences and seminars on personality disorder, and conducted interviews with psychologists, psychiatrists and neuroscientists working in forensic mental health.

My analysis is inspired by the tradition of material-semiotic theory within STS, which foregrounds the relationships between discourse, practice and things in the production and stabilization of science and society (Haraway, 1991; Jasanoff, 2004; Latour, 1987; Pickering, 1995). Such scholarship refuses both (techno)scientific and social reductionism, and instead emphasizes how knowledge, artefacts and social processes mutually constitute and legitimate one another. In my analysis, I was concerned with how the texts in my sample framed (AS)PD. By employing the ‘frame’ metaphor, I draw on Erving Goffman’s (1986) frame analysis, which concerns how experience is socially organized and meaning is ascribed to entities and events, as well as historian Charles Rosenberg’s (1992) argument that, while there is a material and normative quality to disease, the recognition and naming of diseases are social processes. Rosenberg’s scholarship is less ‘programmatically charged’ (Rosenberg, 1992: xv) than other writings on the ‘social construction’ of health and disease, as he refuses to assume that medical institutions are necessarily oppressive. His historical project attempts to remain sensitive to the polysemy of disease across time and space; by doing so, Rosenberg seeks to analyse how particular frames come to be used, rather than appraising how well they fit their ‘object’. Following Goffman and Rosenberg, I am concerned with the framing of personality disorder, and not with the ontological status of the disorders, per se.

With these conceptual and methodological issues in mind, we turn now to the analysis itself, which I introduce with an account of the legal and clinical configurations from which debates about the new MHA emerged. I then summarize some of the policy developments through the late 1990s and early 2000s and discuss the associated perspectives of psychiatrists and psychologists. I follow this with an analysis of the clinical debates about personality disorder that were touched off by the proposed legislative changes, before describing how the policy machinery itself shifted gear in order to accommodate some of the concerns of mental health professionals. The rapprochement between political/policy and clinical goals came to be most visible in four new ‘Dangerous and Severe Personality Disorder (DSPD) Units’, which sought to treat patients with personality disorder, while at the same time managing the risks they presented to the public. Finally, I analyse the culmination of these various debates: the new 2007 MHA, and the growing (though still not universal) belief within the UK mental health professional community that personality disorders are treatable.

By presenting my historical narrative in this way, I seek to demonstrate how debates around, first, mental health law and policy, and, second, clinical knowledge concerning a controversial diagnostic label, became (re)energized and shaped one another. Such an account underscores the great extent to which UK clinicians mobilized powerfully to contest and eventually influence the new MHA, eventually structuring the regimes within which they would have to practice. Accordingly, the making of new mental health law in England and Wales did not entail some kind of unidirectional ‘capture’ of clinical ‘interests’. Rather, as this paper will show, a more complex series of reciprocal interactions and mutually constitutive processes between clinical knowledge, law and policy occurred. While my focus is explicitly on the UK, and on personality disorder, the broad conceptual claim I make – that interactions between medicine and law reframe pathology in important ways – should not be regarded as necessarily restricted to this particular case. Many other nations have debated the moral and clinical issues associated with psychopathy and related conditions, and mental health law is frequently used and critiqued as a means of managing ‘dangerous’ and ‘risky’ individuals. Future comparative work may usefully reveal how different legal and clinical cultures (and the interactions between them) produce different understandings of pathology (cf. Jasanoff, 2005: 15).

## The 1983 MHA and the ‘patients psychiatrists dislike’

In 1983, the first new MHA in more than 20 years was unveiled in England and Wales. This Act, refining and extending much of the content of a 1959 predecessor, was largely steered by the recommendations of an expert committee chaired by Lord Butler of Saffron Walden. These recommendations were widely perceived to be humane, liberal and progressive, and the Act they helped to constitute was favourably received.^3^ However, this is not to say that there was no contention associated with the new Act. The criminological valence that had long been attached to certain aspects of mental health law drew attention, particularly with regard to the administration of offenders who were considered to come within the remit of the Act. The new MHA continued a tradition of allowing courts to order the compulsory detention of offenders with ‘mental impairment’ or ‘psychopathic disorder’. However, it also implemented new restrictions on involuntary hospitalization of criminals (as well as non-offenders) diagnosed with psychopathy.

Specifically, the 1983 Act introduced what came to be known as the ‘treatability test’. This mandated that an offender could only be held in National Health Service (NHS) settings if treatment was available, which was ‘likely to alleviate or prevent a deterioration of his condition’. This ‘test’ ensured that a diagnosis of psychopathy was not by itself sufficient for involuntary hospital detention. Rather, in order to justify legal detention, the disorder had to be defined as treatable. The new treatability test came to direct the attentions of mental health professionals working under the Act to the ontology of psychopathy. In particular it enjoined the question: could psychopaths be treated?

In the UK, as well as in many other national contexts, the treatability of psychopathy had long been a matter of contention.^4^ A minority position, which maintained that therapy was both possible and potentially efficacious, has always been a part of clinical discourse; yet the majority of psychiatrists and psychologists were pessimistic about whether psychopathy could be treated. Following the new Act, forensic psychiatrist Adrian T. Grounds succinctly expressed this pessimism:

The detention of offenders in the legal category ‘psychopathic disorder’ in special hospitals for treatment raises a number of critical issues. There are doubts about the nature of the disorder; what constitutes treatment; who is ‘treatable’; the effectiveness of treatment; and whether evidence of psychological change implies reduced risk of reoffending. (Grounds, 1987: 474)

The treatability test in the MHA invited reflection on these issues, as did the influential third edition of the US nosology, the *Diagnostic and Statistical Manual of Mental Disorders* (DSM-III), published in 1980. DSM-III devoted a major section to personality disorders, and the significance accorded to them was not lost on British psychiatrists. The new category of antisocial personality disorder (ASPD) was particularly interesting to forensic practitioners, due to its apparent overlap with the older category of psychopathy. Furthermore, it had a more specific definition than the opaque construct of psychopathic disorder in the 1983 MHA. Consequently, British practitioners increasingly favoured ASPD over psychopathy for categorizing antisocial individuals.

Previously, psychiatrists had commonly believed that personality disordered individuals were a nuisance, who were far from being proper subjects of the ‘clinical gaze’. Rather, mental health professionals felt that dealing with personality disorder was an impediment to the ‘real work’ of treating people with discrete mental illnesses.^5^ Individuals living under the label of personality disorder were frequently excluded from health services and often were explicitly disliked by practitioners. This disdain was exemplified in an influential 1988 paper, ‘Personality disorder: The patients psychiatrists dislike’ (Lewis and Appleby, 1988). The authors reported results from a study that examined psychiatrists’ attitudes towards personality disorder, and noted that patients characterized with these disorders were described as ‘manipulative, attention-seeking, annoying’ (Lewis and Appleby, 1988: 45).

Clinical pessimism was not universal, however: some psychiatrists conceptualized personality disorders as treatable conditions. An influential proponent of this view was Peter Tyrer, a frequent writer on personality disorder and, from 2003, editor of the *BJP*. In 1991, Tyrer and colleagues, uncomfortable with prejudices against personality disorder, argued that:

One of the important consequences of better classification and awareness of personality problems is the recognition that people with personality disorders suffer considerably and merit help, even if it cannot always be given in a reliable and effective form. In the past, many therapeutic disciplines have tended to regard personality disorders as not really part of psychiatry’s province and that they should therefore be separated from ‘real’ mental illness. This view is often implicit and rarely finds its way into print but is unfortunately common in practice. Views of treatment are now changing. Psychotherapy in particular, which has always maintained that personality disorders are part of its territory, has persevered in attempts to understand and modify the harmful attitudes that dominate the personal lives and relationships of people with personality disorders, and has helped to transfer this awareness to others. (Tyrer et al., 1991: 468)

Tyrer attempted, in one move, to de-stigmatize personality disorders and advocate their treatment, emphasizing both the subjective distress suffered by patients and the extent to which they could be ameliorated though psychotherapy. Nevertheless, treatment could be ‘long, arduous and difficult to complete’ (p. 468). Others agreed.^6^

As inspiring as Tyrer’s comments might have been to some mental health professionals, many others were concerned about the evidence for such claims. In the 1990s, the concept of evidence-based medicine began to resonate powerfully throughout psychiatry, and ‘hard data’ on treatments for personality disorder became more compelling than rhetoric. As a 1998 editorial asserted, ‘[t]here is a need for claims of therapeutic success within the field of personality disorder to be rigorously appraised’ (Cawthra and Gibb, 1998: 8). Such comments echoed those made by psychologist Bridget Dolan and psychiatrist Jeremy Coid in an influential 1993 review of treatments for personality disorders commissioned by the DH and the Home Office. While Dolan and Coid (1993) expressed cautious optimism about treatability, they also emphasised the need for statistically robust trials to provide firm evidence for the effects of such treatment. These longstanding but relatively subdued tensions regarding the treatability of personality disorder were brought into sharp relief at the close of the 20th century when proposals were put forward to revise the 1983 MHA.

## The making of dangerous and severe personality disorder

In 1999, the DH and the Home Office began to develop the new mental health policy. Consequently, clinical attention shifted profoundly toward these government departments. On 15 February 1999, Home Secretary Jack Straw introduced a new phrase to British health professionals: Dangerous and Severe Personality Disorder (DSPD).^7^ The concept of DSPD was widely regarded as having been animated, in part, by the July 1996 murder of Lin and Megan Russell by Michael Stone. A prior-offender diagnosed with a personality disorder, Stone had used a variety of services through the 1990s and was involuntarily admitted into De La Pole Hospital, Hull, in November 1994. He was subsequently discharged in January 1995, following a decision that he was no longer eligible for compulsory detention under the MHA (South East Coast Strategic Health Authority, 2006). Media constructions of a dangerous individual abandoned by mental health professionals as a consequence of legal constraints sat alongside broader public fears about predatory paedophiles and serial killers, and policy-makers appeared pressed to respond to these concerns (Freestone, 2005; Manning, 2002; Prins, 2007; White, 2002). For Straw, it was clear that there was:

a group of dangerous and severely personality disordered individuals from whom the public at present are not properly protected, and who are restrained effectively neither by the criminal law, nor by the provisions of the Mental Health Act … . [T]he government proposes that there should be new legislative powers for the indeterminate, but reviewable detention of dangerously personality disordered individuals. … The individuals concerned must have the best possible chance of becoming safe, so as to be returned to the community, whenever that is possible. (Straw, 1999, quoted in Gunn, 2000: 336)

Emerging from Whitehall rather than the Royal College of Psychiatrists, DSPD was not a medical diagnosis; rather, it was a new administrative category for risky individuals. Specifically, it included psychopaths and others meeting criteria for ASPD who were also believed to present a clear and enduring danger to the public. The law has long sought to control ‘risky’ or ‘dangerous’ individuals (Bartlett, 2003), and not just in the UK. However, what is pertinent here is that the already rather broad mandate of the State for defining and managing dangerousness was considered too narrow by Straw; he felt that powers for indeterminate detention should be extended, regardless of whether the individual to be detained was currently before the courts. The DSPD proposals therefore represented a radical step by the Government to regulate the administration of mentally disordered offenders, and hence of the mental health professions.

In July 1999, a consultation paper was released that advanced the possibility of a legislative change that would enable individuals categorized with DSPD to be subject to indeterminate confinement in a special unit within a prison or NHS hospital (Home Office and the Department of Health, 1999). As ethnographer Mark Freestone (2005: 450) put it, the DSPD Units were intended to be ‘a “third way” between the prison service and the special hospital and as such a unique environment’: a place to detain individuals who were considered to be threats to the public, while at the same time providing mental health care to reduce the risk they presented.

As an instrument of governance, the DSPD proposals were closely related to the Dutch *Ter Beschikking Stelling* (TBS) System (de Boer et al., 2008), an institutional arrangement for the management of mentally disordered offenders, which aimed to reduce their risk to society. The proposed DSPD Units also echoed earlier suggestions made in 1975 by the Butler Committee (McCallum, 2001), and bore a striking resemblance to a recommendation advanced in the so-called Fallon Report (Fallon et al., 1999). Released in January 1999, the Fallon Report detailed the results of an inquiry into drug use, pornography and possible paedophile activity in Ashworth Special Hospital. The report proposed the establishment of special units for individuals with severe personality disorder within prisons and NHS facilities – similar, therefore, to Straw’s DSPD Units, and perhaps an inspiration for them (Bartlett, 2003).

Straw’s plans were unveiled at a time when significant doubts remained about the treatability of personality disorder. With such doubts and the Stone case in mind, the DSPD Programme was an attempt to remedy the social and clinical problems surrounding individuals with severe personality disorders. However, the DSPD proposals were widely resented and resisted by clinicians, and a number of opinion-pieces in well-regarded journals attempted to rally mental health practitioners against them.^8^ In particular, these practitioners raised serious concerns about the possibility that psychiatry would move further from a therapeutic regime, and toward one of public protection.

The strongly authoritarian aspects of the DSPD proposals thus ensured that they would be strongly contested by mental health practitioners, many of who were – following decades of criticisms by ‘anti-psychiatrists’ and others – keenly aware of and anxious about their potential to be agents of social control. However, the DSPD Programme was not the only change to mental health policy and practice that the government sought to introduce: as the 20th century closed, plans were in place to create an entirely new MHA for England and Wales.

## Changing the terrain of mental health

Toward the end of the 1990s, it was increasingly felt by the Government that the 1983 MHA was in need of ‘updating’. Accordingly, in September 1998 they charged an expert committee (chaired by Genevra Richardson, Professor of Law at King’s College, London) to review the Act. The Richardson Committee’s proposals were considered humane and progressive, and were received favourably by clinicians (Department of Health, 1999). For instance, the Richardson Report proposed to further what was widely understood to be the ‘clear therapeutic ethos’ of the 1983 MHA by extending the conditions of the treatability test (Glover-Thomas, 2006: 23; Grounds, 2001).

Four months after the release of the Richardson Report, in November 1999, a government Green Paper was published: **‘**Reform of the Mental Health Act 1983: Proposals for consultation’ (Secretary of State for Health, 1999). Like the DSPD proposals, this detailing of the Government’s effort to reform the 1983 MHA was roundly disliked by clinicians (Zigmond, 2001). This reaction was exacerbated by the fact that, unlike the Richardson Report, the emphasis in the Green Paper was on compulsion – compulsory treatment and compulsory detention.

Further resentment was engendered by the Green Paper’s rejection of many of the Richardson Committee proposals (Bartlett, 2003). For example, in marked contrast to the extension of the treatability test recommended by Richardson, the Green Paper advocated the removal of that test altogether. Furthermore, under its terms, personality disordered individuals could be detained involuntarily on the basis of risk, irrespective of whether the specific DSPD proposals came to fruition.^9^

One point of agreement between the Green Paper, the Richardson Report, the DSPD consultation paper and the Fallon Report was that psychopathy should be removed from the MHA and replaced with the more general category of personality disorder. This was a move deemed by many to be ‘long overdue’ (Laing, 2000: 223), given that, in practice, the legal definition of psychopathic disorder was applied to a range of sub-categories of personality disorder.

Nevertheless, the Green Paper was heavily criticized by clinicians, as well as by members of the Richardson Committee itself.^10^ As lawyer Nicola Glover-Thomas put it, in the Green Paper, ‘risk management has trumped therapeutic endeavour’ (Glover-Thomas, 2006: 32). Though the 1983 MHA referred to the risk presented by a patient, the Green Paper placed this theme in the foreground (Laing, 2000). The emphasis on risk – and the controversy that attracted – characterized the debate on the rewriting of the 1983 MHA over subsequent years.

## The danger of dangerousness

Notwithstanding the negative reaction to the 1999 Green Paper, the Government continued with its plans to revise the 1983 MHA and implement the DSPD Programme. In 2001, £126 million had been committed to DSPD service development, and the programme was being piloted at specialized units at Rampton Hospital and Her Majesty’s Prison (HMP) Whitemoor.

Rather than becoming the authoritarian and illiberal initiative initially feared by clinicians, once it was implemented, the DSPD Programme represented a ‘watered down’ version of the original proposals and worked within the ambit of existing legislation. The Units were extremely well resourced, with little expense spared in terms of both their construction and function.^11^ High costs were justified in the context of the government’s longer-term plans for mental health and crime control.

DSPD also formed the explicit focus of a 2000 mental health White Paper (Department of Health, 2000a,b). Underscoring the Government’s concerns with personality disorder and dangerousness, the White Paper was very poorly received. One *BJP* editorial argued it to be a ‘profoundly illiberal document’ (Grounds, 2001: 387), noting its inclusion of potentially broader criteria under which mentally disordered offenders could be indeterminately detained. Similarly, a *British Medical Journal* editorial (Szmukler, 2001) expressed the continuing concerns of clinicians with the Government’s proposals, and highlighted the difficult balance between care provision and risk control.^12^

Critical voices emanated from across the UK, including professionals at the ‘coal face’ of practice – mental health lawyers, as well as clinicians (Peay, 2003). Critics focused on the preoccupation with risk and ‘dangerousness’ that characterized the White Paper (Buchanan and Leese, 2001); further, they raised questions about the meaning of dangerousness: How would it be measured? How dangerous would an individual have to be to qualify for the DSPD programme? How did dangerousness relate to treatability and to risk? In 2002, a Draft Mental Health Bill was published, but these key questions remained largely unanswered.

Surprisingly, given the Government’s previous focus on DSPD, the 2002 Draft Bill did not specifically address this category. However, mental health stakeholders found the Bill troubling for a variety of reasons, not least because its wording raised concern that highly antisocial individuals (i.e. individuals who might come under the rubric of DSPD) could nevertheless more easily be detained involuntarily within mental health services. Significant debate reigned over this point (Pilgrim, 2007). Yet, as lawyer Peter Bartlett pointed out, the concept of dangerousness was nothing new to English and Welsh mental health legislation (or, indeed, to that of other nations), and contemporary concerns about social control ignored the uncomfortable fact that psychiatry had always played a prominent role in the governance of deviance. Furthermore, it was ‘not obvious’ how far the new Bill extended existing powers for the detention of individuals who were perceived to be a threat to others (Bartlett, 2003: 328). In spite of these caveats to the profusion of clinical concerns, Bartlett nevertheless considered the Bill ‘badly flawed’ (p. 327) and observed that with it, ‘the government managed to achieve a consensus rarely seen in mental health politics. Sadly, the consensus was negative: virtually no one supported the draft bill’ (p. 326).^13^

## Towards treatability

As debate about the MHA reigned, deliberations regarding the treatability of personality disorder became increasingly apparent. Promissory discourse about the potential to treat personality disorder in more efficient and efficacious ways was instantiated within new academic units, such as the Nottingham Personality Disorder Institute, and informal networks and formal associations, such as the British and Irish Group for the Study of Personality Disorder (BIGSPD; formed in 2000). These centres and fora arose from and further animated clinical interest in the aetiology, development and treatment of personality disorder.

At the same time, public spending on personality disorder services and research from bodies such as the MRC, the DH and the DSPD Programme significantly increased. Established in April 1999 (3 months before the release of the initial joint Home Office/DH proposals for DSPD), the National Forensic Mental Health R&D Programme commissioned research on behalf of the DH; in doing so, it became a key funder of scientific and clinical research into personality disorder. The Programme also commissioned literature reviews into the aetiology of personality disorder and its management. Of course the proliferation of research and the promise of new treatments for personality disorder did not readily foster a unified front of clinical optimism; views remained mixed.^14^ Yet, while some clinicians were pessimistic, a growing number of others wrote articles and gave commentaries that pointed towards effective interventions. The London-based ‘therapeutic communities’ at Cassel and Henderson Hospitals were highly regarded for their effective work with individuals with personality disorder (Manning, 2002), and psychotherapeutic strategies more broadly were viewed with optimism, partly as a consequence of a new, growing evidence base.^15^

In particular, faith in psychological techniques such as cognitive analytic therapy (CAT) and dialectical behaviour therapy (DBT, an approach based on cognitive behaviour therapy (CBT)) was increasing during the first few years of the 21st century, even as the number of randomized controlled trials (RCTs) remained low. Pharmaceuticals, such as antipsychotics and antidepressants, were also being used as ‘adjuncts’ to the more conventional psychotherapeutic management strategies for personality disorders, which brought into sharp relief the ontological plurality so often evident within psychiatry (Helén, 2011; Pickersgill, 2010).^16^

Optimism was exemplified in bold claims about treatability. For influential Broadmoor-based psychotherapist Gwen Adshead, there could be ‘no justification for global assertions that personality disorder is untreatable’ (Adshead, 2001: 412). Treatment strategies also became increasingly nuanced. Clinicians were urged to forego the notion of a ‘fix all’ for personality disorder, and to concentrate instead on the ‘functional assessment’ of the condition (Davidson, 2002). The functional approach produced an increasingly modularized view of personality; first, by cataloguing an individual patient’s specific ‘abnormal’ personality features and exploring the distress the patient experienced as a result of them, and, second, by using the catalogued features as the basis for a complex management plan. Such a plan would be ‘bespoke’, rather than ‘off the peg’; individualized, but nevertheless drawing on an eclectic array of standard interventions.^17^ By the end of the 20th century, then, a number of key actors were beginning to treat personality (disorder) as potentially plastic – capable of responding to the appropriate and skilled application of psychiatric and psychological knowledge.

However, despite the enthusiasm that many health professionals expressed toward the idea that personality disorder could be effectively treated, not everyone was so positive. For example, Rampton Hospital psychologist Kevin Howells and his colleagues suggested that different models of personality disorder lay beneath the assorted treatment strategies, raising questions about appropriateness and efficacy (Howells et al., 2007). There also were concerns that many of the treatments touted for a broad spectrum of personality disorder had shown evidence of effects only for borderline personality disorder (Crawford, 2007) – like ASPD, this was one specific ‘variant’ of personality disorder. Furthermore, as Peter Bartlett wryly observed, the ‘predicted treatability of a given psychopath seems to a significant degree dependent on the psychiatrist engaged in diagnosis’ (Bartlett, 2003: 328). Thus, while the mental health professions undoubtedly were moving toward an understanding of personality disorder as treatable, there was by no means a consensus on the issue. Yet it was precisely because the tide of opinion on personality disorder was so clearly turning that some commentators felt compelled to advance such caveats.

## A policy push

While some clinicians at the coalface of practice wrote positively, yet cautiously, about the treatability of personality disorder, there was markedly less subtlety in some of the proclamations made by the DH. In an attempt to correct what was still the common attitude that individuals with personality disorder should not be the concern of mental health services, the newly formed National Institute for Mental Health in England (NIMHE) published a report (National Institute for Mental Health in England, 2003a) that argued that these conditions were treatable; any remaining doubt about this was regarded as lamentable, and something that a new MHA might redress. Indeed, it was explicitly stated that the controversial removal of the 1983 treatability test would ‘highlight the need for new community and in-patient services for people with personality disorder’ (National Institute for Mental Health in England, 2003a: 28). Clinicians were encouraged to be more open-minded to the potential of treatment in light of this.

Ten months later, the NIMHE released the ‘Personality disorder capabilities framework’. This was part of a document provocatively titled ‘Breaking the cycle of rejection’ (National Institute for Mental Health in England, 2003b), and aimed ‘to challenge the discriminatory association between personality disorder and dangerousness by putting in place services aimed at reducing vulnerability and promoting more effective coping by individuals’ (National Institute for Mental Health in England, 2003b: 10). The document further promoted a vision of treatability by painting a more sympathetic picture of personality disorder than many others (including the DH) had in the past:

In recent years, the emphasis on risk and dangerousness associated with a very small number of people with personality disorder, has obscured the fact that very many people with this diagnosis are highly vulnerable to abuse and violence themselves – and to self-harm and suicide. (National Institute for Mental Health in England, 2003b: 10)

Perhaps unsurprisingly, although the NIMHE document alluded to the MHA in its section on ‘The broader policy context’, it refrained from more explicit articulation of the interconnections between the assumed clinical antipathy towards personality disorder and the proposed MHA (specifically, the provision that would remove the treatability test). The controversial category of DSPD – which was at the forefront of many clinicians’ attention at the time – was not even mentioned. The influence of both DSPD and the 2002 Draft Mental Health Bill was nevertheless evident in the list of competencies deemed necessary for clinicians working with individuals diagnosed with personality disorder: in particular, skills in ‘assessing and managing risk to self and others’. Risk, the leitmotif of the proposed reforms to the 1983 MHA, thus structured the NIMHE clinical guidelines, even as the same document made gestures to de-stigmatize the disorder.

The assumed importance of a consensus that personality disorder was treatable to the acceptance of the removal of the treatability test was also instantiated through a 2003 treatment review (Warren et al., 2003). The review, which was commissioned by the Home Office and DH through the DSPD Programme, aimed to provide an evidence base for ‘informing the decisions about the development of services for DSPD’ (Warren et al., 2003: 8). Building on the aforementioned review by Dolan and Coid (1993) (Bridget Dolan was one of the authors of the 2003 report), the 2003 ‘update’ came to similar conclusions: namely, that personality disorder was (potentially) treatable, especially through the application of therapies such as DBT and therapeutic community models. There was markedly less evidence for pharmacological management strategies, not least because of the allegedly poor methodological design of published studies – a limitation perceived to be characteristic of much of the existing research.

Unfortunately, Warren and colleagues could find no RCTs – the biomedical ‘gold standard’ – within the most secure NHS facilities. This deficiency was lamented by Warren et al., although they nevertheless argued that their findings were substantiated by including less ‘reliable’ studies in their review (Warren et al., 2003: 5). By doing so, they could make the mass of evidence supporting the claim that personality disorder was treatable seem weightier than if they had employed rigorous statistical inclusion criteria. In short, the review contained a message that both policymakers and many clinicians were receptive to: personality disorder was treatable, but more research was needed.

In sum, through 2002 and 2003, the development of policy and clinical discourse on personality disorder brought the treatability of these conditions sharply into focus. In recognition of the controversy it had generated and mindful of its aims to successfully implement DSPD policy, the DH sought to ally itself with mental health professionals. It did this, first, by evoking tropes similar to those that had become increasingly evident within clinical discourse – chiefly, those tropes that tended to de-stigmatize personality disorder and to emphasize its treatability. Second, it articulated these tropes within the pages of key documents, which aimed, in part, to foster a more favourable outlook towards the proposed new mental health legislation. In the process, the hopes of clinicians were brought more closely into alignment with the aims of policymakers.

## How to treat dangerousness

The alignment of policy and clinical goals was most markedly apparent in the new DSPD Units. By autumn 2005, four high security pilot DSPD Units were running, which contained ‘some of the most difficult and challenging individuals in society’ (DSPD Programme, 2005: 27). Two Units were inside prisons (HMP Frankland and HMP Whitemoor), and two were within NHS hospitals (Broadmoor and Rampton). Generally speaking, the prison Units stressed the correctional aspects of the DSPD Programme, whereas the hospitals focussed more on the therapeutic components. However, even within DSPD Units, there were tensions between carceral and clinical approaches (Freestone, 2005; Maltman et al., 2008)

Treatment itself, at least in Broadmoor and Rampton Hospitals, was administered by multidisciplinary teams consisting of clinical and forensic psychologists, psychiatrists and mental health nurses, working together with occupational therapists and other health and social care professionals. These individuals acted simultaneously to implement the DSPD policy and to treat the psychological and social deviancy of patients diagnosed with personality disorder. Treatment aimed to reduce offending behaviour, though many clinicians were concerned to treat ‘the person in personality disorder’, rather than managing only those aspects that were (in the modularized vision of personality) assumed to be associated with (if not determinative of) offending behaviour.

While medication was occasionally used as an adjunct, psychological therapies were the principal tools used by mental health professionals in the DSPD Units.^18^ In particular, CBT (of the sort used in the probation service) and related techniques predominated.^19^ As Armstrong (2002) has shown, CBT is well positioned to serve both punitive/criminological and therapeutic/clinical ends: it is retributive (individuals are held accountable for their antisocial behaviour) while also being rehabilitative (antisocial behaviour decreases following therapy). Accordingly, it is not surprising that CBT migrated from probation services to the criminological–medical hybrid that is the DSPD Programme.

The role and nature of treatment in DSPD Units draws our attention to three central issues. First, DSPD Units are sites where the goals of policymakers and clinicians are closely aligned: policymakers can be satisfied that the risk that dangerous offenders represent is being reduced, while psychiatrists and psychologists are given the time and resources to obey a clinical imperative to treat individuals diagnosed with personality disorder. Second, the autonomy of clinicians within DSPD Units complicates the presumption of the unilateral political capture of clinical goals that might be inferred from the previous section. That some health professionals practising within the DSPD Programme frame their work as treating personality disorder rather than solely managing the risky behaviours associated with it reveals a more dynamic, though still asymmetric, relationship between the aspirations of the DH and clinical communities. Third, the activities of the DSPD Units have subtle but potentially profound implications for the understanding of personality disorder. With few established conventions regarding the ‘correct’ way to treat these conditions but with a remit to do so regardless, professionals in the DSPD Units experimented with different forms of psychotherapy and medication. The various treatments relied on diverse underlying models of personality disorder; by juxtaposing these therapies, heterogeneous models of the conditions were assembled and reconstructed. The DSPD Units therefore acted as laboratories within which framings of personality disorder could be experimented with, and they played a salient role in the constitution of the conception that personality disorder was treatable.

## The social shaping of mental health law

Though clinicians were busy experimenting with personality in the DSPD Units, they nevertheless found time to continue contesting the Government’s ever-evolving plans for mental health law. As we have seen, the 2002 Draft Mental Health Bill did not garner the support the DH had hoped. It was thus withdrawn and, in September 2004, a new Bill was released (Department of Health, 2004a). Somewhat less controversial than its predecessor, the Bill marginally mollified detractors by reintroducing some treatability criteria for cases where involuntary compulsion was being considered. Despite this move, it was still criticized for its emphasis on risk. Furthermore, some of its detractors claimed that the Bill ignored, for the most part, the views of important mental health institutions such as the Royal College of Psychiatrists. This was interpreted as exemplifying the Government’s indifference both to the clinicians who would implement the new MHA and the patients who would be under its purview (Brown, 2006).

The Government, however, considered that it had ‘taken seriously the concerns raised about the first version of the draft Bill’ (Department of Health, 2004b: 4), but it did not push forward the second Bill. Seemingly in response to the massive criticism levelled against it over the previous seven years, the DH instead made a different move; specifically, it announced in March 2006 that, rather than completely rewriting the 1983 MHA, it would instead amend it (Brown, 2006). Accordingly, on 18 November 2006 a (third) new Mental Health Bill was released (Department of Health, 2006a).

The 2006 Bill was, of course, criticized. Like its predecessors, it forsook the treatability test; according to the DH, this was because the test had led ‘to a false presumption that some – particularly those with severe personality disorders – are untreatable. This means that detention is sometimes not used when it ought to be, even though people with severe personality disorders can be – and are – treated compulsorily under the Act’ (Department of Health, 2006b: 3). By removing the treatability test, the legislation arising from the 2006 Bill would ‘take away some unnecessary obstacles to practitioners’ ability to use the Act where it is warranted by the needs of the patient and the degree of risk’ (Department of Health, 2006b: 3).

Yet, the treatability test was not altogether abandoned. In its place stood the new ‘appropriate treatment test’. Similar to the 1983 treatability test, this revised set of criteria for detention included the key qualification that treatment should be readily ‘available’ for patients, rather than merely being a theoretical possibility. Apparently, the appropriate treatment test would call for ‘an holistic assessment of whether appropriate treatment is available’ (p. 2); this was seen as a means of shifting clinical attention away from treatment outcomes alone – a perceived flaw of the 1983 treatability test.

Nevertheless, what, precisely, a ‘holistic assessment’ entailed was unclear, so too was the procedure for establishing (or formally contesting) whether a particular treatment was ‘appropriate’. Furthermore, many commentators were concerned that the Bill illegitimately conflated ‘appropriate’ with ‘effective’ treatment. In other words, they were concerned that individuals could be detained as a consequence of *some* kind of treatment being available, irrespective of whether it was the *right* kind. By removing the *treatability test* and replacing it with the *appropriate treatment test*, the DH sought to ensure that people who ‘had’ to be involuntarily detained could be held, but with appropriate safeguards in place: at least some kind of treatment needed to be available for the individual being detained. Accordingly, it was argued that the new legislation would not result in ‘any significant increase in people detained’ (p. 3).

In spite of the concerns outlined above, eventually the Bill was passed with some modifications. Thus, after almost a decade of political wrangling, a new MHA for England and Wales was given Royal Assent on 19 July 2007. In marked contrast to the original plan for a radical reconstruction of the 1983 Act, the new MHA was an amended version of its predecessor. The chief detractors of the DH were, for the most part, mollified (Mental Health Alliance, 2007); not surprisingly, though, some disapproval still was registered (Prins, 2007).

## Personality disorder is treatable (isn’t it?)

On 22 June 2007, one month before the 2007 MHA received Royal Assent, the National Forensic Mental Health R&D Programme officially closed. The DH, it seems, no longer felt the need to continue to invest so significantly in research concerning the treatability of personality disorder, since effective treatments were now widely considered available.^20^ As Frankie Pidd, of the DH National Personality Disorders Development Programme, commented in an article co-authored with psychologist Janet Feigenbaum:

The evidence base has emerged over the past two decades to indicate that personality disorders are treatable. A range of psychological therapies has been shown to be the most effective treatment for personality disorders, though medication can have some additional effect in reducing the severity of symptoms. (Pidd and Feigenbaum, 2007: 8)

Though Pidd and Feigenbaum accepted that some clinicians might disagree, they asserted that such disagreement was based on a ‘false belief’ (Pidd and Feigenbaum, 2007: 7), and, worse, a belief that would increase the stigmatization of individuals diagnosed with personality disorder. Pidd and Feigenbaum therefore evoked a narrative of de-stigmatization, consistent with the trend in the mental health professions since at least the 1990s (Pilgrim and Rogers, 2005), which was especially apparent in recent years in the discourse concerned with personality disorder. In so doing, Pidd and Feigenbaum presented not only a therapeutic imperative to work with individuals with personality disorder, but an ethical duty as well.

Further, journals such as *Psychology, Crime and Law* devoted entire issues to contributions on personality disorder, many of which newly emphasized the treatability of these conditions. As widely regarded clinical psychologist Peter Fonagy noted in one special issue’s introduction, clinicians should ‘celebrate the emergence of effective biological and psychosocial treatments’ (Fonagy, 2007: 3). For personality disorder expert John Livesley, the literature was now ‘clear that personality disorder can be treated’ (Livesley, 2007: 28).^21^

It can be seen, then, that claims in recent years to the effect that personality disorder is treatable have come to resound powerfully through clinical discourse: reported inside journal pages, argued through presentations in large conferences and small seminars, and attested to by practising psychologists and psychiatrists. Today, individuals with disordered personalities are still considered problematic, ‘one of the most difficult groups in psychiatric practice’ (Newton-Howes et al., 2006: 18). But many professionals now at least consider that treatment is possible, despite the continued scepticism.

## Conclusion

This article has shown how personality disorders came to be widely regarded as treatable in the clinical professions and the policy arena. In particular, I have highlighted the important role of mental health policy debates in helping to effect this shift. In significant ways, attempts to rewrite the 1983 Mental Health Act of England and Wales directed new attention to longstanding problems with personality disorder: How should offenders with this diagnosis be managed? Who should take responsibility? Are conditions like ASPD and psychopathy even treatable? While law is a powerful tool for ordering a society, debates over legislative changes cast into sharp relief the ambiguities law is designed to manage. By proposing to rewrite the MHA – in particular, by issuing forth highly controversial and contested proposals – the DH animated clinical discourse on personality disorder, creating new fora for clinicians to articulate their views on the treatability of these conditions. Such debate was also directly fostered by the DH; for instance, when it commissioned reviews on and research into treatments. In time, a new convention began to emerge: personality disorder was framed by many clinicians as treatable, and personality itself was regarded as plastic; an entity mouldable through the skilful application of clinical technique. Therapeutic technologies did not so much involve the use of novel pharmaceuticals; instead, they made use of more familiar psychological methods.

Visions of treatability were instantiated within the new DSPD Units, which themselves straddled the boundaries between the mental health and criminal justice systems. The Units, which were set up by the State as part of plans to reform the management of mentally disordered offenders, achieved legitimacy through the emerging consensus among clinicians that personality disorders such as ASPD and psychopathy were now treatable. The very existence of the Units arguably helped to legitimate this novel accord, and, in turn, to justify the vast public expenditure on the contested and controversial category of the population held in those Units. Political intentions to remove the treatability test from the 1983 MHA seemed more palatable after individuals with personality disorder were widely understood to be patients who could – and should – benefit from clinical intervention; the dismay expressed by many mental health professionals about changes to the MHA were damped down. The ‘danger’ of DSPD was, to an extent, neutralized.

While the DH profited from the new understanding of personality as a treatable entity, so too did clinicians who had long held that belief. The material and symbolic benefits for these psychologists and psychiatrists included acceptance by their peers, as well as increased funding, jobs, and support for treating patients with personality disorder, particularly through the DSPD Programme (Manning, 2002). Clinicians used the resources of DSPD Units to treat their patients, while at the same time they critiqued the notion that DSPD was a legitimate medical category. In this way clinicians, in part, appropriated and subverted the very policy aimed at their regulation.^22^

More generally, we have seen how UK clinicians mobilized broadly and powerfully to contest and eventually influence the new MHA, and that they succeeded in shaping, if not determining, a law that would, in time, govern their practice. The recent history of British mental health is not, therefore, a simple story of the linear ‘capture’ of clinical goals by policymakers. Rather, it is a more complex narrative about the mutual constitution of policy and practice, of legal structures and clinical knowledge, and of the multiple acceptances and resistances that have enabled this.

The DSPD Units became important sites for treatment and research, and therefore influential hubs, in the new medical-legal network that resulted from the dynamics outlined in this paper. Within these sites, clinicians conducted trials of diverse treatments that implied different developmental mechanisms for personality disorder, and thereby reconstituted ideas about what these conditions were and how they might be acted upon. In effect, psychiatrists and psychologists rebuilt the DSPD Units as laboratories for experimenting with all aspects of personality disorder, and thus with understandings of personality itself. This exemplifies the potential of local sociotechnical spaces – themselves synthesized through heterogeneous assemblages of institutions, discourse and practice – to play a central role in the generation of clinical knowledge (in this case, knowledge of the nature of personality disorder and how to manage it).^23^

As we have seen, the mutual constitution of mental health policy and practice has profoundly altered understandings of personality disorder. Conditions such as ASPD and psychopathy are no longer largely considered obdurate, and are now often framed as potentially plastic, mouldable through clinical intervention. This reconceptualization on the part of a number of forensic mental health practitioners has not led to a panacea: not everyone believes that personality disorder can be treated, and doubts remain about the future of DSPD policies and practices, including the decommissioning of Units.^24^ But a significant shift has occurred, as a direct consequence of the reciprocal interaction between clinical and policy debates, and the mutual shaping of knowledge and law. And for many, psychopaths will never be the same again.

## Notes

1. For more on the history and sociology of personality disorder, especially ASPD and psychopathy, see: Bendelow (2010), Greig (2002), Gurley (2009), Lane (2009), Manning (2000, 2002, 2006), McCallum (2001), Pickersgill (2009a,b; 2010; 2011a,b; 2012a,b), Pilgrim (2007) and Werlinder (1978). As these authors show, framings of personality disorder have mutated over the last 150 years or more, and diverge and converge in different ways between nations.

2. Northern Ireland and Scotland have separate legislation.

3. See Moncrieff (2003), Simpson (1976) and Wootton (1980).

4. In particular, debates around the treatability of psychopathy in Australia, Canada, the Netherlands and the US have connected with discussions in the UK. Many commentators across North America and Western Europe have interrogated similar questions to those raised in Britain.

5. As discussed by Cavadino (1989).

6. See, for example, Stein (1992), Stone (1993) and Yorston (1999).

7. Or ‘Jack Straw Syndrome’, as DSPD became colloquially known among psychiatrists such as Peter Tyrer (2001) who were critical of these developments.

8. See for example: Chiswick (1999), Eastman (1999), Freestone (2005), Haddock et al. (2001), Mullen (1999) and White (2002).

9. The homogenization of the category ‘personality disorder’ has long been common in UK mental health, in spite of attempts by formal diagnostic handbooks (or, rather, those who produce them) to instantiate sub-categories. The Green Paper accepted this, and indeed explicitly rejected the definitional schemes of the US DSM-IV. Instead, it sought a unified definition of ‘mental disorder’. Accordingly, even though ASPD and psychopathy over-shadowed the other forms of personality disorder, so long as an individual was labelled as both severely personality disordered and dangerous, and their dangerousness was regarded as causally linked to their personality disorder, then that person could – in theory – be involuntarily detained. In this sense, the government seized on established convention within UK mental health (that is, to regularly speak of personality disorder broadly, rather than specific sub-types) in order to introduce proposals that were widely regarded as illegitimate.

10. In particular, see Peay (2000) and Szmukler and Holloway (2000).

11. The 70-bed Peaks Unit at Rampton Hospital alone cost £20 million to build (Nottinghamshire Healthcare NHS Trust, 2004) and the management of individual DSPD prisoners/patients came to cost ‘around £300,000 per annum’ within hospitals, and ‘around £85,000 per place per annum’ within prisons (of which ‘about £35,000 p.a.’ was ‘related to treatment costs’) (Department of Health/National Offender Management Services Offender Personality Disorder Team, 2011: 12).

12. See also Chiswick (2001) and Gunn and Felthous (2000).

13. See also Moncrieff (2003: 8).

14. See, for example, Chiesa et al. (2000), Haddock et al. (2001) and Kendell (2002).

15. See, for example: Bateman and Fonagy (1999), Chiesa and Fonagy (2000) and Mullen (1999).

16. Treatment is discussed by Sushovan and Tyrer (2001) and Triebwasser and Siever (2007).

17. The logics and implementation of these ‘bespoke’ programmes are outlined by Hogue et al. (2007) and Livesley (2007).

18. Psychological therapies are also emphasized in UK clinical practice guidelines as treatments for both ASPD (National Institute for Health and Clinical Excellence, 2009) and borderline personality disorder (National Collaborating Centre for Mental Health, 2009).

19. For discussion, see Maden (2005) and de Boer et al. (2008).

20. Of course, research continues around personality disorder treatments. It is certainly not my aim to suggest that no research was or is taking place on personality disorder outside of the National Forensic Mental Health R&D Programme, but rather to underscore that research conducted within that programme commanded particular attention.

21. These developments followed further state interest in treatments for personality disorder. The DSPD Programme continued and expanded: by 2008, alongside the original four high-secure units, four medium secure units and some community services for DSPD were in operation (de Boer et al., 2008).

22. As other scholars have shown, psychiatric attitudes and ideology can strongly influence mental health policies, which, in turn, can shape clinical work and opinion (Grob, 2008; Prior, 1991; Schatzman and Strauss, 1966)

23. Though of course they were not the only sites of research into personality disorder.

24. See, for instance, Department of Health/National Offender Management Services Offender Personality Disorder Team (2011).
